# Granulocyte Colony-Stimulating Factor in Amniotic Fluid

**DOI:** 10.1155/S1064744995000482

**Published:** 1995

**Authors:** B. Denise Raynor, Penny Clark, Patrick Duff

**Affiliations:** 1 Division of Maternal Fetal Medicine Department of Obstetrics and Gynecology University of Florida Gainesville FL USA; 2 Department of Gynecology and Obstetrics Emory University School of Medicine Glenn Building, 69 Butler Street SE Atlanta GA 30303 USA

## Abstract

*Objective:* The purpose of this study was to determine if granulocyte colony-stimulating factor (G-CSF) is normally present in amniotic fluid and then to determine if amniotic-fluid G-CSF levels are affected by labor and intrauterine infection.

*Methods:* Amniotic fluid was collected from 35 patients in 4 groups: no labor, early labor, late labor, and labor plus chorioamnionitis. G-CSF levels were measured by enzyme-linked immunosorbent assay (ELISA).

*Results:* The mean amniotic-fluid G-CSF concentrations prior to labor were lower than during labor (0.49 ± 0.25 ng/ml for prior to labor vs. 1.83 ± 1.0 ng/ml for labor, *P* < 0.001). With chorioamnionitis, the mean levels were elevated compared with normal labor (25.0 ± 4.8 ng/ml for chorioamnionitis vs. 1.83 ± 1.0 ng/ml for normal labor, *P* < 0.0001). In early and late labor, G-CSF was higher than prior to labor (0.49 ± 0.25 ng/ml for no labor vs. 1.48 ± 1.0 ng/ml for early labor, *P* < 0.02, vs. 2.2 ± 0.8 ng/ml for late labor, *P* < 0.0005). The mean concentrations in early and late labor were not different.

*Conclusions:* G-CSF is present in amniotic fluid and increased with labor. When labor is complicated by chorioamnionitis, G-CSF is significantly elevated.


Infectious Diseases in Obstetrics and Gynecology 3:140–144 (1995)
© 1995 Wiley-Liss, Inc.

### Granulocyte Colony-Stimulating Factor in Amniotic Fluid

**B. Denise Raynor, Penny Clark, and Patrick Duff**

*Division of Maternal Fetal Medicine, Department of Obstetrics and Gynecology, University of Florida, Gainesville, FL*

### Abstract

***Objective:*** **The purpose of this study was to determine if granulocyte colony-stimulating factor (G-CSF) is normally present in amniotic fluid and then to determine if amniotic-fluid G-CSF levels are affected by labor and intrauterine infection.**

***Methods:*** **Amniotic fluid was collected from 35 patients in 4 groups: no labor, early labor, late labor, and labor plus chorioamnionitis. G-CSF levels were measured by enzyme-linked immunosorbent assay (ELISA).**

***Results:*** **The mean amniotic-fluid G-CSF concentrations prior to labor were lower than during labor (0.49 ± 0.25 ng/ml for prior to labor vs. 1.83 ± 1.0 ng/ml for labor, $P < 0.001$). With chorioamnionitis, the mean levels were elevated compared with normal labor (25.0 ± 4.8 ng/ml for chorioamnionitis vs. 1.83 ± 1.0 ng/ml for normal labor, $P < 0.0001$). In early and late labor, G-CSF was higher than prior to labor (0.49 ± 0.25 ng/ml for no labor vs. 1.48 ± 1.0 ng/ml for early labor, $P < 0.02$, vs. 2.2 ± 0.8 ng/ml for late labor, $P < 0.0005$). The mean concentrations in early and late labor were not different.**

***Conclusions:*** **G-CSF is present in amniotic fluid and increased with labor. When labor is complicated by chorioamnionitis, G-CSF is significantly elevated.** © 1995 Wiley-Liss, Inc.

Key words

Intrauterine infection, labor, growth factors, chorioamnionitis

Granulocyte colony-stimulating factor (G-CSF) is a hematopoietic growth factor that stimulates the differentiation of neutrophils and enhances neutrophil function. It acts at several sites: stimulating the proliferation of progenitor cells in bone marrow, accelerating the differentiation of mature forms, and enhancing chemotaxis and phagocytosis through priming of the cellular metabolism associated with oxidative burst. G-CSF is synthesized by fibroblasts, endothelial cells, macrophages, placental cells, and decidua.

At birth, the host defenses in neonates are immature. Frequently, newborns respond to sepsis with neutropenia because of low mature-neutrophil storage and myeloid progenitor-cell pools. In addition, when compared with adults, mature neonatal neutrophils have qualitative deficiencies in neutrophil activation, chemotaxis, phagocytosis, and bacterial killing.

Recombinant G-CSF (rG-CSF) has been shown to cross the placenta and decrease neonatal mortality in experimentally induced sepsis in animal models.[1,2] The maternal administration of rG-CSF to improve neonatal survival from peripartum infection has recently been considered. However, the data on G-CSF concentrations in normal newborn serum are conflicting, with some studies showing minimal amounts of the stimulating factor and others showing elevated amounts compared with

**Address correspondence/reprint requests to Dr. B. Denise Raynor, Department of Gynecology and Obstetrics, Emory University School of Medicine, Glenn Building, 69 Butler Street SE, Atlanta, GA 30303.**

**Presented at the 15th Annual Meeting of the Society of Perinatal Obstetricians, Atlanta, GA, January, 1995.**

Received September 19, 1995
Accepted September 20, 1995

adults.[3–5] Little information is available on serum levels of G-CSF in infected neonates.

The source of G-CSF in amniotic fluid is unclear. While placental macrophages and decidual cells synthesize this factor, the fetus may contribute to amniotic-fluid concentrations, especially when infected. Both the presence and source of G-CSF in amniotic fluid may have some bearing on the efficacy of the intrapartum administration of rG-CSF.

We sought to determine the normal levels of G-CSF in amniotic fluid and any change in these levels during labor and in the presence of chorioamnionitis.

### SUBJECTS AND METHODS

The study was conducted at Shands Hospital, University of Florida, Gainesville, between April and June of 1994. Our institution serves primarily a rural, indigent patient population. This study was approved by the institutional review board. A signed consent form was not required in the protocol because the collection of amniotic-fluid specimens posed no risk to the patient or her fetus. However, verbal permission was obtained from each patient prior to the amniotic-fluid collection.

Amniotic fluid was collected from patients in 4 groups selected at random based on the availability of the investigators: 1) those not in labor, 2) those in early labor (defined as ≤4-cm cervical dilation), 3) those in late labor (defined as near delivery, after complete cervical dilation), and 4) those in labor with clinical chorioamnionitis. Chorioamnionitis was diagnosed by the physician caring for the patient if she developed a fever ≥38°C in association with maternal or fetal tachycardia and uterine tenderness in the absence of any other localizing signs of infection. The amniotic-fluid specimens were collected after the diagnosis was made without regard to cervical dilation. A patient was excluded from the first 3 groups if she developed the signs of chorioamnionitis after the specimen was collected. No patient received amnioinfusion prior to the specimen collection. The amniotic fluid was collected by needle aspiration at the time of the uterine incision during a cesarean delivery or by aspiration through an intrauterine pressure catheter that had been placed for the usual obstetrical indications. The amniotic fluid was aspirated from the pressure catheter using a sterile syringe. The initial 10 cc of fluid was discarded, and the next 10–15 cc was aspirated into a fresh sterile syringe, which was capped after expelling the air and transported to the research laboratory within 30 min of collection.[6]

The amniotic fluid was centrifuged at 3,900*g* for 10 min; the supernatant was collected and stored at −80°C. None of the samples was meconium-stained. G-CSF was measured using duplicate samples by commercial enzyme-linked immunosorbent assay (ELISA) (R&D Systems, Minneapolis, MN). The double antibody sandwich assay used a murine monoclonal anti-G-CSF antibody as the capture antibody and a polyclonal anti-G-CSF conjugated to horseradish peroxidase as the detecting agent. G-CSF concentrations were determined by reference to a standard curve of known concentrations of human rG-CSF. The assay had no cross reactivity with interleukin-1 (IL-1), IL-2, IL-3, IL-6, IL-8, tumor necrosis factor (TNF), or granulocyte-macrophage colony stimulating factor (G-CSF). The lower limit of detection was 15 pg/ml. The mean of the 2 measurements was used when analyzing the results. The intra-assay coefficient of variance was 5.5%.

The maternal age and parity, gestational age at delivery, mode of delivery, duration of ruptured membranes, interval between specimen collection and delivery, and use of oxytocin were recorded.

The differences in categorical variables were analyzed with the corrected chi-squared test. The differences between continuous variables were assessed by the Mann-Whitney U-test for non-normally distributed variables such as G-CSF levels and the 2-tailed, paired t-test for normally distributed variables such as gestational age and duration of labor. The correlation was performed by Spearman rank test. $P < 0.05$ was considered statistically significant.

### RESULTS

The study group consisted of 35 patients: those not in labor (7), those in early labor (9), those in late labor (9), and those in labor with chorioamnionitis (10). The complications of pregnancy included postdates (2) and preterm delivery (1) in the 2 laboring groups; previous cesarean (3), fetal macrosomia (2), and preeclampsia (2) in the no labor group; and spontaneous premature rupture of the membranes (2), preeclampsia (2), polyhydramnios (1), and gestational diabetes (1) in the chorioamnionitis group.

The use of oxytocin and the method of delivery

**TABLE 1. Use of oxytocin and method of delivery in study patients**

| Group (no.) | Oxytocin | Cesarean |
|---|---|---|
| Early labor (9) | 8 | 1 |
| Late labor (9) | 8 | 1 |
| Chorioamnionitis (10) | 9 | 7 |
| Prelabor (7) | — | 7 |

are detailed in Table 1. Thirteen (37%) of the 35 amniotic-fluid specimens were collected at the time of cesarean: 5 in patients with chorioamnionitis, 1 late in labor, and 7 prior to labor. The indications for cesarean delivery in the study population were arrest of labor (7), previous cesarean (3), nonreassuring fetal heart rate tracing (2), malpresentation (2), and macrosomia (2). All but 2 of the intrauterine pressure catheters were placed to monitor oxytocin administration, either for augmentation or induction of labor. One catheter was inserted because of an inadequate tocodynameter recording and 1 for an amnioinfusion for variable decelerations.

The mean gestational ages were 36 ± 4 weeks for patients without labor, 40 ± 2 weeks for those in early labor, 38 ± 4 weeks for those in late labor, and 38 ± 3 weeks for those in labor with chorioamnionitis. These differences were not statistically significant. The 2 patients without labor who delivered preterm infants account for the lower mean gestational age in that group. Both had preeclampsia.

In the early labor group, samples of amniotic fluid were collected, a mean of 6 ± 4 h prior to delivery, significantly longer than the 1 ± 1 h in the late labor group ($P < 0.001$), but not longer than the 2 ± 3 h in the group with chorioamnionitis. The patients with chorioamnionitis had a significantly longer interval with ruptured membranes, 13 ± 7 h, compared with those in the early and late labor groups, 8 ± 4 and 6 ± 3 h, respectively ($P < 0.01$). The infected patients were more likely to be delivered by cesarean ($P < 0.005$).

The mean G-CSF concentrations in the patients prior to the beginning of labor were significantly lower than those in labor (0.49 ± 0.25 ng/ml for prelabor vs. 1.83 ± 1.0 ng/ml for labor, $P < 0.001$) (Fig. 1). In patients with chorioamnionitis, the mean amniotic-fluid levels were significantly elevated compared with those in labor (25.0 ± 4.8 ng/ml for chorioamnionitis vs. 1.83 ± 1.0 ng/ml for labor,

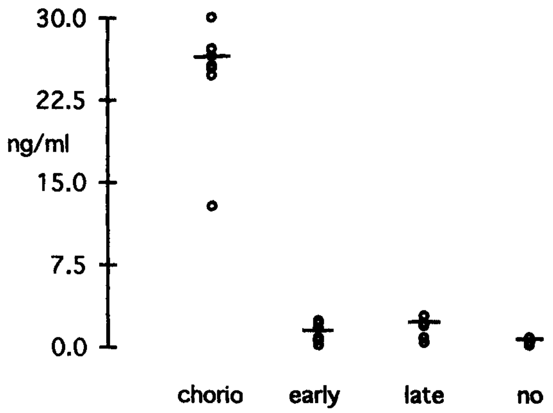

**Fig. 1. Amniotic-fluid G-CSF levels in chorioamnionitis, early labor, late labor, and prior to labor. Bars represent means.**

$P < 0.00001$) and prior to labor (0.49 ± 0.25 ng/ml, $P < 0.00001$). While mean G-CSF levels in both early and late labor were significantly higher than prelabor (0.49 ± 0.25 ng/ml for prelabor vs. 1.48 ± 1.0 ng/ml for early labor, $P < 0.02$, vs. 2.2 ± 0.8 ng/ml for late labor, $P < 0.0005$), there was no difference between early and late labor. However, there was a significant correlation between G-CSF levels and the interval between the specimen collection and delivery ($R = 0.50$, $P < 0.05$); i.e., G-CSF levels tended to rise as labor continued. G-CSF levels correlated with neither gestational age nor cervical dilation, even when the significantly higher levels in the chorioamnionitis group were excluded.

### DISCUSSION

G-CSF is a major modulator of neutrophil function, affecting both the number and activity of these cells. Our study showed that G-CSF is present in the amniotic fluid at term and increases with the duration of labor. In the presence of clinical chorioamnionitis, a 10-fold elevation in amniotic-fluid G-CSF levels occurs. This increase presumably reflects the increase in WBCs seen in infected amniotic fluid. These findings support those of Saito et al.,[7,8] who reported increased amniotic-fluid G-CSF levels in women in labor and a further increase when histologic evidence of chorioamnionitis was identified or bacterial endotoxin was present in the amniotic fluid. Our study patients were somewhat different from those in the previous study since histologic chorioamnionitis and amniotic-fluid bacterial endotoxin can be seen in subclinical intraamniotic infection without the overt signs of clinical chorioamnionitis.

A number of the shortcomings in our study should be mentioned. While the women included in the laboring groups had essentially uncomplicated pregnancies, some pregnancies in the chorioamnionitis and prelabor groups were complicated by preeclampsia and gestational diabetes. The question of whether either of these diseases has an effect on G-CSF concentrations is unexplored. The similarity of the specimens of G-CSF concentrations in the presence of infection would suggest that infection is the major stimulus for G-CSF secretion. It is also possible that our sample of patients in labor may not be representative of normal labor since the majority of laboring patients received oxytocin. However, all but 2 were delivered vaginally, suggesting that the labor patterns ultimately normalized. Lastly, our study was limited by the small sample size.

The source of amniotic-fluid G-CSF is as yet unclear. Decidual macrophages have been shown to synthesize G-CSF, while cytotrophoblasts and stromal cells do not appear to produce this stimulating factor.[9] However, immunohistochemical studies have localized G-CSF to decidual stromal cells and trophoblasts,[8] and receptors have been demonstrated on placental membranes and trophoblasts.[10]

Nonpregnant adults have undetectable or low serum G-CSF levels which rise significantly with acute infection.[11] Information on serum concentrations in pregnancy is limited to a small sample which showed no correlation between maternal serum levels and infection, although G-CSF was elevated in one-fifth of the mothers studied.[14]

Little is known about fetal G-CSF production. While Laver et al.[3] reported higher G-CSF concentrations in term neonates than adults, neither Cairo et al.[5] nor Bailie et al.[4] found high stimulating factor levels in umbilical cord blood from term neonates.[3–5] The latter study found preterm neonates had elevated G-CSF concentrations when compared with term infants, but Gessler et al.[12] reported that neonates between 26 and 37 weeks gestation had lower cord blood levels than those born after 37 weeks. The neonates born to mothers with signs of maternal infection had 4-fold higher G-CSF levels soon after birth than those born to uninfected mothers, and the newborns who developed actual infections had a 7-fold increase compared with those without infections. The elevated G-CSF levels, however, did not result in a statistically significant increase in the total neutrophil count.

G-CSF mRNA production by mononuclear cells from both umbilical cord blood and adult blood has been shown to be negligible. With stimulation, G-CSF mRNA expression was lower in mononuclear cell cultures from neonates than adults and was even lower in cells cultured from preterm neonates.[5,13] This finding may explain the lower serum G-CSF concentrations in neonates than adults. Further studies are needed to determine the basal fetal production of G-CSF, the maternal and fetal contributions to the concentration of G-CSF in amniotic fluid, and the way in which these factors may affect fetal and neonatal responses to sepsis.

### REFERENCES

1. Medlock ES, Kaplan DL, Cecchini M, Ulich TR, del Castillo J, Andresen J: Granulocyte colony-stimulating factor crosses the placenta and stimulates fetal rat granulopoiesis. Blood 81:916–922, 1993.
2. Novales JS, Salva AM, Modanlou HD, Kaplan DL, et al.: Maternal administration of granulocyte colony-stimulating factor improves neonatal rat survival after a lethal group B streptococcal infection. Blood 81:923–927, 1993.
3. Laver J, Duncan E, Abboud M, Gasparetto C, et al.: High levels of granulocyte and granulocyte-macrophge colony-stimulating factors in cord blood of normal full-term neonates. J Pediatr 116:627–632, 1990.
4. Bailie KE, Irvine AE, Bridges JM, McClure BG: Granulocyte and granulocyte-macrophage colony-stimulating factors in cord and maternal serum at delivery. Pedriatr Res 35:164–168, 1994.
5. Cairo MS, Suen Y, Knoppel E, Dana R, et al.: Decreased G-CSF and IL-3 production and gene expression from mononuclear cells of newborn infants. Pediatr Res 31: 574–578, 1992.
6. Gibbs RS, Blanco JD, St. Clair PJ, Casteneda YS: Quantitative bacteriology of amniotic fluid from women with clinical intraamniotic infection at term. J Infect Dis 147: 1–8, 1982.
7. Saito S, Kato Y, Ishihara Y, Ichijo M: Amniotic fluid granulocyte colony stimulating factor in preterm and term labor. Clin Chim Acta 208:105–109, 1992.
8. Saito S, Kasahara T, Kato Y, Ishihara Y, Ichijo M: Elevation of amniotic fluid interleukin 6 (IL-6), IL-8 and granulocyte colony stimulating factor (S-CSF) in term and preterm parturition. Cytokine 5:81–88, 1993.
9. Shorter SC, Vince GS, Starkey PM: Production of granulocyte colony-stimulating factor at the materno-foetal interface in human pregnancy. Immunology 75:468–474, 1992.

10. Uzumaki H, Okabe T, Sasaki N, et al.: Identification and characterization of receptors for granulocyte colony stimulating factor on human placenta and trophoblastic cells. Proc Natl Acad Sci USA 86:9323–9326, 1989.
11. Kawakami M, Tsutsumi H, Kumakawa T, et al.: Levels of serum granulocyte colony stimulating factor in patients with infection. Blood 76:1962–1964, 1990.
12. Gessler P, Kirchman N, Kientsch-Engel R, Haas N, Lasch P, Kachel W: Serum concentration of granulocyte colony stimulating factor in healthy term and preterm neonates and in those with various diseases including bacterial infections. Blood 82:3177–3182, 1993.
13. English BK, Hammond WP, Lewis DB, Brown CB, Wilson CB: Decreased granulocyte-macrophage colony-stimulating factor production by human neonatal blood mononuclear cells and T cells. Pediatr Res 31:211–216, 1992.
